# Antagonistic selection on body size and sword length in a wild population of the swordtail fish, *Xiphophorus multilineatus*: Potential for intralocus tactical conflict

**DOI:** 10.1002/ece3.7288

**Published:** 2021-03-20

**Authors:** Melissa N. Liotta, Jessica K. Abbott, Molly R. Morris, Oscar Rios‐Cardenas

**Affiliations:** ^1^ Department of Biological Sciences The Ohio Center for Ecological and Evolutionary Studies Ohio University Athens OH USA; ^2^ Department of Biology Lund University Lund Sweden; ^3^ Instituto de Ecología AC Red de Biología Evolutiva Xalapa, Veracruz México

**Keywords:** alternative reproductive tactics, genetic conflict, intralocus tactical conflict, tactical dimorphism, tactically antagonistic selection

## Abstract

Alternative reproductive tactics (ARTs) have provided valuable insights into how sexual selection and life history trade‐offs can lead to variation within a sex. However, the possibility that tactics may constrain evolution through intralocus tactical conflict (IATC) is rarely considered. In addition, when IATC has been considered, the focus has often been on the genetic correlations between the ARTs, while evidence that the ARTs have different optima for associated traits and that at least one of the tactics is not at its optimum is often missing. Here, we investigate selection on three traits associated with the ARTs in the swordtail fish *Xiphophorus multilineatus*; body size, body shape, and the sexually selected trait for which these fishes were named, sword length (elongation of the caudal fin). All three traits are tactically dimorphic, with courter males being larger, deeper bodied and having longer swords, and the sneaker males being smaller, more fusiform and having shorter swords. Using measures of reproductive success in a wild population we calculated selection differentials, as well as linear and quadratic gradients. We demonstrated that the tactics have different optima and at least one of the tactics is not at its optimum for body size and sword length. Our results provide the first evidence of selection in the wild on the sword, an iconic trait for sexual selection. In addition, given the high probability that these traits are genetically correlated to some extent between the two tactics, our study suggests that IATC is constraining both body size and the sword from reaching their phenotypic optima. We discuss the importance of considering the role of IATC in the evolution of tactical dimorphism, how this conflict can be present despite tactical dimorphism, and how it is important to consider this conflict when explaining not only variation within a species but differences across species as well.

## Graphical abstract

We investigated two of the key criteria necessary to demonstrate intralocus tactical conflict (IATC): whether alternative reproductive tactics (ARTs) have different phenotypic optima for a shared trait and whether one or both ARTs were not at their phenotypic optimum for a shared trait. We addressed these criteria estimating selection on body size, body shape, and sword length (elongation of the caudal fin) in the swordtail fish *Xiphophorus multilineatus*. Our study suggests that IATC is constraining both body size and the sword from reaching their phenotypic optima.

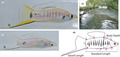

## 1 INTRODUCTION

Studies of the evolution of sexual dimorphism have led to many insights into the evolutionary mechanisms that can produce phenotypic diversity within a species (Andersson, 1994; Darwin, 1871; Lande, 1980). Sexually antagonistic selection, different selective optima between males and females for a shared trait due to their different life histories, is a main driver in the evolution of sexual dimorphism (Cox & Calsbeek, 2009; Mank, 2009; Rice, 1984). However, the evolution of sexual dimorphism can be constrained if the trait is genetically correlated between the sexes. Intralocus sexual conflict occurs when males and females have different optima for genotypes at a given locus (Abbott et al., 2010; Arnqvist & Rowe, 2005; Bonduriansky & Chenoweth, 2009; Bonduriansky & Rowe, 2005; Chippindale et al., 2001; Long & Rice, 2007). If unresolved, intralocus sexual conflict can generate gender load, reduction of the population's mean fitness due to the displacement of one or both of the sexes from their phenotypic optima (Bedhomme & Chippindale, 2007). While research on intralocus conflict has mainly focused on the sexes, theory from intralocus sexual conflict is readily applicable to other polymorphisms. In particular, within‐sex polymorphisms such as alternative reproductive tactics (ARTs) have only recently been considered in the context of intralocus conflict (Abbott et al., 2019a, 2019b; Abbott & Svensson, 2010; Buzatto et al., 2015; Morris et al., 2013; Pennell et al., 2018; Pike et al., 2017).

Alternative reproductive tactics (ARTs) are discrete variations in the reproductive phenotypes of members of the same sex within a species (Taborsky & Brockmann, 2010). ARTs often evolve in response to strong sexual selection and involve alternate ways to gain reproductive success through suites of correlated behavioral, morphological, and life history traits (Oliveira et al., 2008). Analogous to the sexes, the optimal phenotype for a shared trait often differs between ARTs (Emlen, 1996; Engqvist & Taborsky, 2016; Gross, 1985; Moczek & Emlen, 2000; Taborsky, 2001). Antagonistic selection should drive the evolution of tactical dimorphism, identified as a bimodal distribution of a trait within a sex; however, if the tactics have a shared genetic architecture, then evolution toward their respective optimum can be constrained by intralocus “tactical” conflict (IATC, Morris et al., 2013). The few studies on intralocus tactical conflict have mainly focused on genetic correlations for traits between ARTs (Abbott & Svensson, 2010; Buzatto et al., 2015, 2018; Pike et al., 2017), with much less work on the respective phenotypic optimum for shared traits (Abbott & Gosden, 2009). Studies of intralocus tactical conflict can help us to better understand how ARTs evolve, how they are maintained, and the evolutionary consequences that can arise from the loss of an ART (e.g., speciation). In addition, it will be important to consider the possibility of intralocus tactical conflict when examining the evolution of sexually selected traits across species, in particular if some of the species have alternative reproductive tactics and some do not.

**FIGURE 1:**
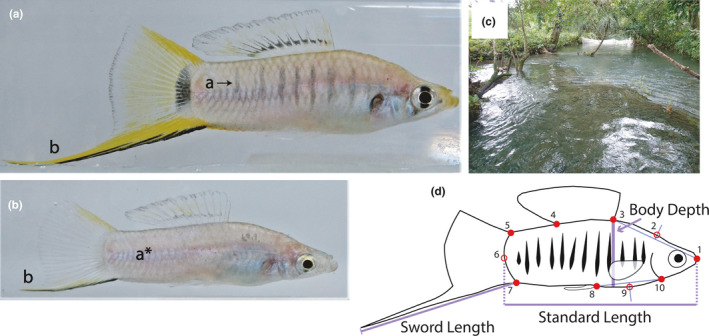
Courter (a) and sneaker (b) males of *Xiphophorus multilineatus*. a, vertical body bars; a*, no vertical body bars present on this sneaker male; b, the sword, extension of the ventral caudal fin rays; (c) sampling for DNA and morphology took place in a side stream of the Río Tambaque located just outside of Aquismón, San Luis Potosí, Mexico. (d) Red filled circles = landmarks; red open circles = semilandmarks; blue line = semilandmark markers or linear measurements; numbers correspond to landmark locations (see Materials and Methods). (a, b photographs by Luke Weinstein; c by Melissa Liotta)

In this study, we investigated the potential for traits associated with the male ARTs in the High‐backed Pygmy swordtail fish, *Xiphophorus multilineatus* (Rauchenberger et al., 1990), to be experiencing antagonistic selection. *Xiphophorus multilineatus* males are classified into two ARTs: larger courter males (Figure 1a) that mature later and are behaviorally fixed (use only a courtship display to attract females); and smaller sneaker males that mature earlier and are behaviorally plastic, using courtship and sneak‐chases when alone with a female, and switching almost exclusively to sneak‐chases when courter males are present (Figure 1b, Zimmerer & Kallman, 1989). While the names of the ARTs in this species are based on the mating behaviors they use, there are a suite of associated traits that are tactically dimorphic, thus with a bimodal distribution (Bono et al., 2011; Liotta et al., 2019; Moretz & Morris, 2003; Morris et al., 2007; Rios‐Cardenas et al., 2018; Zimmerer & Kallman, 1988) including body size, which is genetically influenced by the number of copies of the *Mc4r* gene on the *Y* chromosome (Lampert et al., 2010). The genetic influence on body size suggests that this is a good system in which to examine intralocus tactical conflict. Simulations have been used to demonstrate that for autosomal, *X*‐linked, and *Y*‐linked genetically determined tactics, the estimated intertactical genetic correlations will generally be high (Abbott et al., 2019a). In addition, the presence of tactical dimorphism indicates the potential for IATC since dimorphism between the ARTs is likely evolving due to differing phenotypic optima, and thus net disruptive selection between ARTs (Morris et al., 2013). However, as shown with sexually antagonistic selection and intralocus sexual conflict (Cox & Calsbeek, 2009), the extent of dimorphism may not indicate the extent of conflict. Also, ARTs may not be dimorphic for a shared trait that is experiencing IATC, especially if the dimorphism is slow or difficult to evolve due to genetic correlations.

Using a wild population of *X. multilineatus*, we quantified the strength and mode of selection acting on the ARTs for three morphological traits, allowing us to determine whether the ARTs have different optima for these traits, as well as whether one or both ARTs are not at their optimum. Evidence for these two criteria is necessary for demonstrating intralocus tactical conflict (Morris et al., 2013). Estimating the strength of this conflict will require additional studies that measure the genetic correlation between the ARTs. The first trait we considered was body size, which has previously been shown to be tactically dimorphic in this species (Liotta et al., 2019; Rios‐Cardenas et al., 2018; Zimmerer & Kallman, 1989). Our measure of selection on body size will include mating success of individuals that survived to sexual maturity (Lyons et al., 2014; Morris et al., 2010; Rios‐Cardenas et al., 2007), and not the selection on reaching sexual maturity earlier, which increases smaller males’ probability of reaching sexual maturity (Bono et al., 2011; Morris et al., 2016; Weinstein et al., 2019). Therefore, our results will allows us to tease apart the influence of sexual selection and natural selection on this trait. The second trait we examined was body shape, which has also been shown to be tactically dimorphic in *X. multilineatus*, as well as correlated with the propensity to use sneak‐chase behavior within the sneaker ART (Liotta et al., 2019). The relationship between this trait and mating behaviors within the sneaker ART provides an opportunity to examine the prediction that behavioral plasticity will reduce selection on correlated morphological traits (Abbott et al., 2019b; DeWitt et al., 1998). Body shape in fishes is also well‐documented predictor of swimming performance and locomotive ability (Blake, 2004; Webb, 1984) and could directly impact the successful execution of each ARTs’ respective mating behavior, thus influencing fitness. Body shape also has the potential to function as a sexual signal, with deeper bodies increasing mating success through female mate preference and male–male competition (MacLaren et al., 2004). Finally, the third trait we examined was sword length, an extension of the ventral caudal fin rays, which has not yet been examined in relation to tactical dimorphism in *X. multilineatus* (Figure 1a,b). This trait was first described in the green swordtail, *X. helleri* (Haeckel, 1848), and is considered a hallmark of sexual selection (Darwin, 1871) due to the classical trade‐off between female mate preference for longer swords (Basolo, 1990a, 1990b, 1998) and costs due to natural selection (Basolo & Alcaraz, 2003; Hernandez‐Jimenez & Rios‐Cardenas, 2012; Oufiero et al., 2014; Oufiero, Meredith, et al., 2014). However, evidence for selection on this trait in the wild is surprisingly lacking. The implications for our results are discussed in relation to how intralocus conflict will influence our understanding of the evolution of tactical dimorphism, as well as the overall variation in sexually selected traits between species.

## 2 MATERIALS AND METHODS

### 2.1 Field collection and laboratory conditions

In November 2014, we exhaustively sampled a side stream of the Río Tambaque, San Luis Potosí, Mexico (Figure 1c), for *X. multilineatus* using baited minnow traps and seine nets. The side stream sampled is blocked off from the main branch of the Tambaque by a strip of land and has only one entrance and exit. Water flows in from the main branch of the Tambaque, and then, passing over a shallow ledge meets backup with the main branch. It is easy for fish to enter the side stream and most likely exit out over the ledge; otherwise, the stream is relatively self‐contained. All adult males collected (identified by the presence of a fully developed copulatory organ, i.e., gonopodium) were photographed and fin‐clipped for DNA in the field. Fin clips were stored in 95% ethanol for later DNA extraction. Males were classified as either sneaker (*n* = 44) or courter (*n* = 36) males in the field using standard length (distance from tip of snout to end of caudal peduncle; Figure 1d) and pigmentation patterns, as described by Zimmerer and Kallman (1989). A subset of females (*n* = 47) collected in the stream were brought back to Ohio University to drop fry. Females were isolated in individual 21‐L tanks and kept on a 12‐hr/12‐hr light–dark cycle. Fish were fed twice a day with Ken's Premium Spirulina Max Flake (Ken's Fish Inc.) in the morning and newly hatched brine shrimp nauplii (Brine Shrimp Direct) in the afternoon. Female *Xiphophorus multilineatus* can fertilize eggs from stored sperm for 7 months and can drop a brood every month (Meffe & Snelson, 1989). Females’ tanks were checked daily for fry, and when found, they were collected, euthanized, and preserved in 95% ethanol. Forty‐three out of the forty‐seven females gave birth for a total of 526 fry. After females had exhausted sperm storage, they were fin‐clipped for DNA, and fin clips were stored in 95% ethanol.

### 2.2 Microsatellite genotyping

In order to determine each males’ reproductive success, we conducted a paternity analysis using five microsatellite loci, *KonT38*, *KonD6*, *KonD21*, *KonT30*, and *KonD15* developed for the closely related species *Xiphophorus montezumae* (see Seckinger et al. (2002) for primer sequences and development). These loci have been shown to provide sufficient polymorphisms for assigning paternity in *X. multilineatus* (Luo et al., 2005). Genomic DNA was extracted from the tissue samples (fin clips for adults and half of a fry's whole body) using the Qiagen DNeasy Kit (Qiagen) following the manufacturer's protocol, with the exception that fry whole body tissue was digested overnight and adult fin tissue digested for 3 hr. Fry DNA was eluted at 50 µl and adult DNA at 100 µl. DNA was extracted from and microsatellite loci were amplified for a total of 649 fish (36 courter males, 44 sneaker males, 43 females, and 526 fry). Microsatellite loci were amplified in two multiplex PCRs for each sample (MIX1 = *KonT38*, *KonD6*, *KonD21*, MIX2 = *KonT30*, and *KonD15*). The forward primers were labeled with the fluorescent tags NED (*KonT38*, *KonT30*), VIC (*KonD6*), and 6‐FAM (*KonD21*, *KonD15*). See Appendix S1 for additional details. PCR products were run on a DNA Analyzer using the GeneScan 600 LIZ Size Standard (Applied Biosystems) at the Ohio State University Plant‐Microbe Genomics Facility for fragment analysis. Alleles were scored using Geneious ver. 9.1.8 (Kearse et al., 2012, www.geneious.com).

Microsatellite loci quality was assessed using several programs. We used the GENEPOP ver. 1.0.5 R package (Rousset, 2008) to calculate allele frequencies using the adult samples, whether loci deviated from the Hardy–Weinberg equilibrium (HWE), linkage disequilibrium (LD) among pairs of loci, and null allele frequencies. *p*‐Values for HWE and LD were estimated using the Markov chain method with the following parameters: 1,000 dememorization steps, 5,000 batches, and 1,000 iterations per batch for all tests. Additionally, MICRO‐CHECKER ver 2.2.3 (Van Oosterhout et al., 2004) was used to check for scoring errors. We used CERVUS 3.0.7 (Kalinowski et al., 2007) to check for observed versus expected heterozygosity and exclusion probabilities. When checking through scored genotypes, we noticed mismatches between known dams and offspring. Therefore, dams that did not have at least one allele that matched any offspring and/or offspring in a female's broods that did not have at least one allele that matched their known dam were checked manually for scoring errors in Geneious. In most cases, this issue occurred in individuals that were originally assigned homozygous genotypes, but upon further review, a poorly amplified peak was found that was originally thought to have been a stutter peak. In 1 fry, 3 loci were not amplified, and in 14 fry, one or more loci did not have an allele that could be matched to their known mother. Thus, 15 fry were not included in subsequent paternity analyses, for a total of 511 out of 526 fry included.

### 2.3 Paternity analysis

Paternity was assigned using CERVUS 3.0.7 (Kalinowski et al., 2007). CERVUS uses maximum likelihood to calculate a log‐likelihood ratio score (LOD), which is the likelihood that a male is the true parent of an offspring given the known genotype of the offspring's mother and the offspring's own genotype. A positive LOD score indicates that a male is more likely to be the true sire than not. The program also calculates a “delta” score or the difference in LOD scores between the two most likely candidate sires, which helps in assignment when two males have a positive LOD score for an offspring. In addition, CERVUS uses simulation to estimate a maximum and minimum confidence level in assignment of the most likely sire by taking into account the number of candidate sires (total possible sires in the population both sampled and unsampled), proportion of possible sires actually sampled, completeness of loci typed, and the estimated typing error. In our CERVUS simulation, we chose 0.95 and 0.80 as the maximum and minimum confidence levels, respectively. Simulation parameters were as follows: 10,000 offspring, 92 candidate sires, 87% of sires sampled, 100% of loci typed, and 0.01 error rate. We estimated the number of candidate sires as the average number of males collected in the Río Tambaque over five sampling years and percentage of sires sampled represents the total number of males genotyped (*n* = 80). Assignments with at least an 80% confidence level were kept for further analysis.

### 2.4 Morphometrics and tactical dimorphism

We examined three traits that are either known or suspected to be tactically dimorphic in this species: body size, body shape, and sword length. We first considered widely used linear measurements of these traits (we subsequently refer to these as unidimensional traits). We measured each fish for standard length (Figure 1d) and body depth (pelvic‐fin insertion to dorsal‐fin origin; Figure 1d). Sword length was measured from the insertion point of the ventral caudal fin ray to the distal tip of the ventral ray extension (Figure 1d) using the line tool in ImageJ ver. 1.50i (Schneider et al., 2012). Finally, we also visually confirmed that there was an extension beyond the edge of the caudal fin and that there was pigmentation along the dorsal edge of the caudal fin, given that this aspect of the sword has been identified as important in relation to female mate preference (Basolo, 1995). Unless otherwise indicated, the relations between these unidimensional variables will be controlled statistically with a multiple linear regression (see Selection Analyses below).

We then used landmark‐based geometric morphometrics (Zelditch et al., 2012) and principal component analyses to examine multiple components of body shape, allowing for a more detailed examination of how selection acts on total body shape. Males were photographed in the field using a Canon Power Shot (Canon Inc.), plastic view box with a ruler taped to the front, and a small hand net with the netting material tightened to create a flat surface. Fish were gently pressed against one side of the view box using the net and photographed. The fish's left side was used for landmark placement; if the left side was not photographed, the right side was mirrored so that all individuals faced the same way.

We digitized seven landmarks and three semilandmarks using tpsDig2 (Rohlf, 2015) as per the methods in Liotta et al. (2019): (1) tip of rostrum; (2) forehead; (3) anterior dorsal ray insertion; (4) posterior dorsal ray insertion; (5) dorsal caudal fin insertion; (6) last scale of the midline; (7) ventral caudal fin insertion; (8) anterior insertion of the gonopodium; (9) abdomen; and (10) ventral occlusion of the operculum (Figure 1d; Culumber et al., 2011; Johnson et al., 2014; Liotta et al., 2019). The three semilandmarks were used to characterize the forehead, total body elongation, and the abdomen (points 2, 6, and 9, respectively). Markers were drawn for the forehead and abdomen semilandmarks so that the points could be placed consistently by drawing a straight line between landmarks 1 and 3 and between landmarks 8 and 10 in Adobe Illustrator (Adobe Systems Inc.,) and placing the semilandmark 90° from the midway point (Johnson et al., 2014; Liotta et al., 2019). Landmark coordinate files were obtained using tpsUtil (Rohlf, 2015) and imported into R ver 3.6.0 (R Development Core Team, 2019) for analysis using the package *geomorph* (Adams et al., 2018; Adams & Otárola‐Castillo, 2013). Landmark 4 was estimated for 11 individuals, and landmarks 3 and 4 were estimated for 1 individual using the thin‐plate spline method in the “estimate missing landmark” function in *geomorph* due to the dorsal fin not being extended and obscuring the view of the ray insertion. All analyses were performed in R unless stated otherwise.

As in Liotta et al. (2019), using all males (both courters and sneakers) we performed a generalized Procrustes analysis to remove information unrelated to shape (i.e., size, orientation, and position) by optimal rotation of the coordinates using the least squares criterion, aligning, and scaling to unit centroid size (Rohlf & Slice, 1990). Semilandmarks were slid along their tangent directions using the Procrustes distance criterion (Rohlf, 2015). The aligned coordinates were then projected into tangent space to obtain Kendall's tangent space coordinates (Rohlf, 1999). These shape variables were subjected to a principal component analysis (PCA) using the covariance matrix. All axes that explained at least 10% of the variation were included in further analyses. Centroid size was calculated as the square root of the summed squared distances of each individual landmark to the centroid of the shape (Zelditch et al., 2012).

We determined whether the traits’ standard length, body depth, sword length, sword index (calculated as sword length/standard length to correct for body size), centroid size, and body shape (PC axes 1–4) were tactically dimorphic using a Welch's two‐sample independent *t* test. Standard length, body depth, sword length, and sword index were all log‐transformed for normality. We also considered dimorphism in sword length in relation to extension beyond the caudal fin by comparing the number of males in each ART with and without a sword extension with a Yates‐corrected chi‐square test. In addition, tactical dimorphism in body shape was examined by visualizing the overlap of the 95% confidence ellipses around each group's centroid for each PC axis in relation to PC1.

### 2.5 Selection analyses

We calculated both selection differentials (*s*) and selection gradients for two sets of traits: three unidimensional traits (body size measured as standard length, body shape measured as depth, and sword length), and then the combination of the geometric morphometric trait for body size (centroid), all PC axes explaining >10% variation in body shape, and sword length. Sword length was included in both sets of traits, allowing us to assess the potential of a correlation in selection on this trait and the different measures of body size and body depth.

We calculated each male's absolute fitness (total offspring sired) using the estimates of reproductive success from the paternity analysis. Any male that was not assigned offspring was given an absolute fitness of zero. Relative fitness was calculated as absolute fitness divided by mean fitness across all males. Then, all independent variables were standardized to a mean of zero and unit variance (Lande & Arnold, 1983).

The selection differentials were estimated from linear models with relative fitness regressed on each trait separately, including ART and an interaction between ART and the trait. A significant overall result suggests that at least one of the ARTs is not at its optimum, and a significant interaction with ART suggests the ARTs have different optima. As selection differentials include both direct and indirect selection, we determined whether the selection on the trait was direct by estimating the linear selection gradients (*β*) from the standardized partial regression coefficients of multiple linear regressions. The models included relative fitness as the dependent variable, the respective standardized independent variables measuring body size, body shape, and sword length, a male's ART classification, and two‐way interactions between each trait and ART. A significant *β* coefficient suggests that significant selection detected in the differential analysis is direct, and not due to correlation with another trait in the analysis, while a significant coefficient for the quadratic gradient (*y*_ii_) suggests either disruptive or stabilizing selection. The linear selection gradients (*β*, directional selection) were estimated from multiple regressions that included only linear terms, since if the data violate multivariate normality, the linear and quadratic coefficients will be correlated (Brodie, 1992). The quadratic selection gradients (*y_ii_*, stabilizing or disruptive selection) were estimated from models that included both linear and quadratic terms (Brodie et al., 1995). Quadratic coefficients were doubled as linear models underestimate stabilizing/disruptive selection by half (Stinchcombe et al., 2008). We used marginal effects plots to visualize the significant linear and quadratic selection gradients using the Effects package (Fox & Weisburg, 2018, 2019). We also calculated and reported the selection coefficients for both of the ARTs from each of the full models. For all models, if the interaction term between a trait and ART was significant, subsequent separate models were performed for each ART in order to determine whether the ARTs’ slope differed from zero for that trait. Relative fitness was always calculated from the whole male population.

Violation of the assumption that model residuals are normally distributed is not problematic for estimating the linear and quadratic coefficients; however, violation of this assumption is problematic for estimating statistical significance (Lande & Arnold, 1983). As this was the case for our models, significance values for the selection differentials and gradients were calculated using a resampling procedure in which relative fitness was randomly shuffled across individuals to obtain a null distribution for each gradient where there is no relationship between trait and fitness. Thus, probabilities were the number of times (out of 9,999 permutations) in which the gradient pseudo‐estimate was greater than (if the original gradient was positive) or less than (if the original gradient was negative) the original estimated gradient (Lewis et al., 2011). All statistics were performed in R ver 3.6.0 (R Core Development Team, 2019) unless stated otherwise.

## 3 RESULTS

### 3.1 Microsatellite genotyping

After fixing dam–offspring mismatches when possible, 4 out 5 loci were within expected population genetic parameters in the adult population, that is, within the expectations of HWE, exhibited genetic independence (no significant LD), and had low null allele frequencies. In addition, MICRO‐CHECKER reported no evidence of scoring errors for all 5 loci. However, *KonT38* was problematic for all population genetic parameters. *KonT38* was not within HWE (*p* < .001), it exhibited significant linkage disequilibrium with *KonD15* and *KonT30* (*p* = .007, *p* = .032, respectively), and the estimation for a null allele frequency was considerable (0.171). Therefore, we ran subsequent paternity analyses both with and without *KonT38*. In the absence of *KonT38,* the other four loci again were within expected population genetic parameters.

### 3.2 Paternity analysis

The paternity analysis run with all five loci assigned paternity to 334 out of 511 fry to 51 out of 80 males (28 sneaker, 23 courters) at the minimum 80% confidence level. This is lower‐than‐the‐expected assignment of 416 fry (81% assignment rate) from the simulation, although this is likely due to the null allele increasing mismatches between fry and candidate sires. Runs excluding *KonT38* with four loci were only able to assign 139 fry. Dakin and Avise (2004) report that loci with a null allele frequency equal to or below 0.2 will not greatly affect exclusion probability (in our case it was 0.171). Given that a true parent may be falsely excluded in this case, estimates of reproductive success will likely be underestimated. Therefore, we decided to use the run with all five loci as this is a conservative estimate of paternity. Courter males had higher reproductive success (mean # of fry sired ± *SE*, 6 ± 1.44) than sneaker males (3 ± 0.64). The mean number of fry sired among all males was 4 ± 0.76 fry, with a maximum of 38 fry sired (see Figure S1 for the distribution of paternity).

### 3.3 Morphometrics and tactical dimorphism

Courters and sneakers were dimorphic for standard length (SL) and body depth (BD), with courters on average 11.2 mm longer and 4.5 mm deeper than sneakers (SL: *t = *18.43, *df = *54.56, *p* < .001, BD: *t = *11.18, *df = *64.35, *p* < .001). The swords were 8.6 mm longer on average for courter males as compared to sneaker males (*t* = 12.94, *df* = 61.27, *p* < .001). The sword index, sword length relative to standard length, was also dimorphic, with courter males on average having a 39.71% longer sword per body size than sneaker males (*t* = 6.99, *df* = 57.57, *p* < .001). The swords were also tactically dimorphic in the extent to which they extended beyond the caudal fin: There were more adults with swords that did not extend beyond the caudal fin for sneaker males (30 of 44) than for courter males (0 of 36; Yates chi‐square 36.4, *p* = <.0001). Swords continue to grow after sexual maturity (Basolo, 1990b), and ideally to compare sword length within and across ARTs, models should include total male age and size. In wild‐caught males, it is only possible to assess age up until sexual maturity (using otolith rings), and therefore, we were unable to include total age as a fixed effect in our models. However, assessment of sword length is still informative given we sampled exhaustively for males in this population, which should yield a random distribution of ages.

**FIGURE 2:**
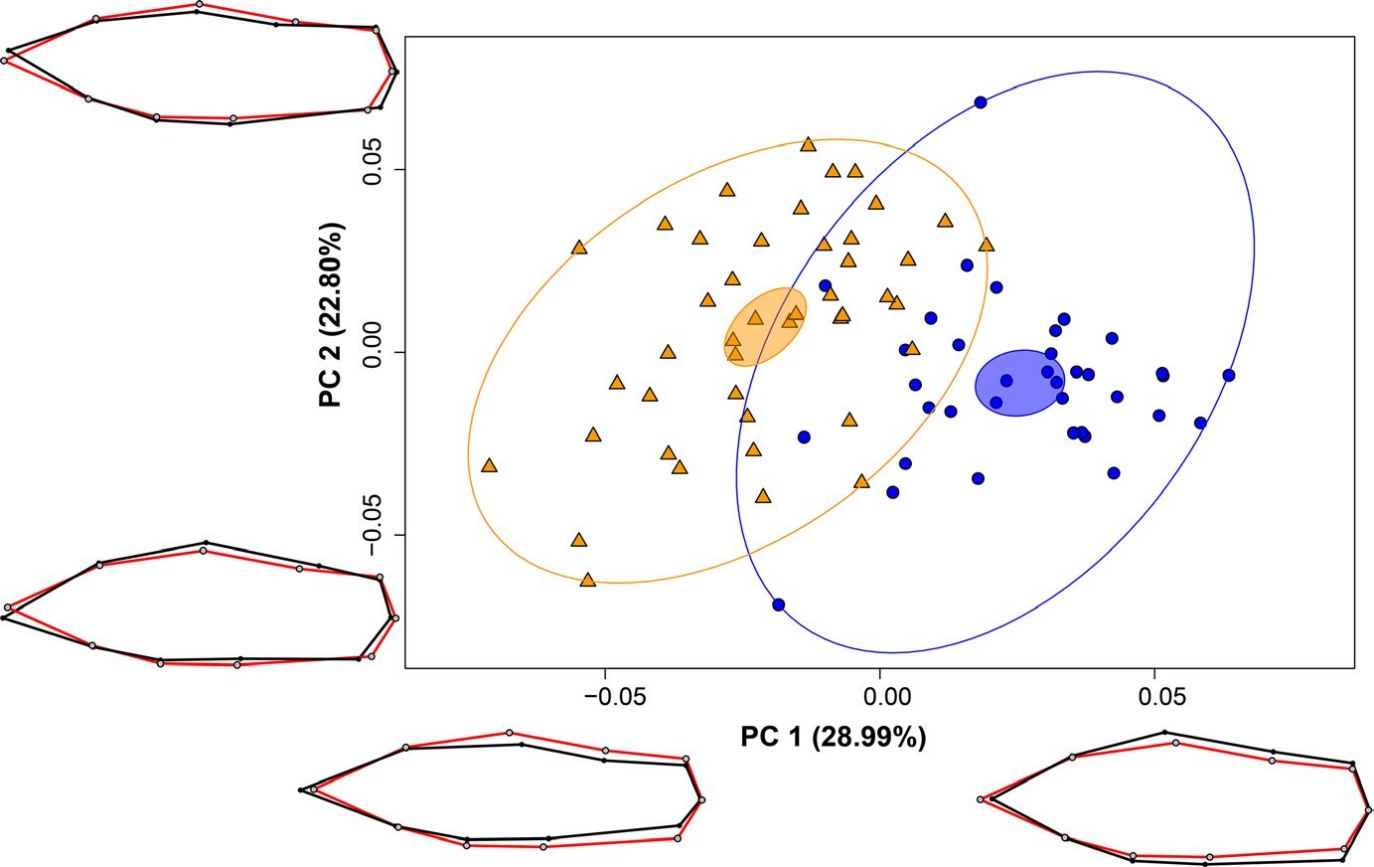
Principal component analysis of the variation in body shape along the first two principal component (PC) axes. Orange triangles = sneaker males; blue circles = courter males. Wire‐frame models representing the extreme minimal and maximal changes in shape along PC1 and PC2 are shown (red = consensus shape, i.e., average body shape of all males; black = shape change, i.e., the change in body shape from the average body shape). Small filled ellipses are the 95% confidence regions around the respective group centroids

Courters and sneakers were tactically dimorphic for centroid size, with courter males being 35.23% larger than sneaker males (*t* = 15.93, *df* = 44.37, *p* < .001). As for body shape, the first four PC axes each explained at least 10% of the variation (PC1 = 28.99%, PC2 = 22.80%, PC3 = 14.81%, and PC4 = 11.40%). Courters and sneakers are dimorphic along the first major axis of variation PC1 (*t* = 10.31, *df* = 75.25, *p* < .001), with courter and sneaker males occupying separate areas of morphospace, with no overlap in the 95% confidence ellipses along this axis (Figure 2). Wire‐frame models generated for each PC axis represent the extreme minimum and maximum shape changes along each axis, and for PC1 suggest sneaker males were overall more fusiform than courter males, which were comparatively more dorsoventrally expanded in their body shape, consistent with findings from Liotta et al. (2019). The ARTs are also dimorphic along PC2 (*t* = −2.68, *df* = 77.64, *p* = .009), which described the variation in the distance between the anterior and posterior dorsal‐fin insertion points, the slope of the forehead (steep or shallow), deepening in the abdomen, and slight elongation or shortening of the caudal peduncle (Figure 2). However, courters and sneakers are not dimorphic along PC3 (*t* = −1.15, *df* = 65.20, *p* = .254) and PC4 (*t* = −0.906, *df* = 77.89, *p* = .368).

### 3.4 Selection analyses

**FIGURE 3:**
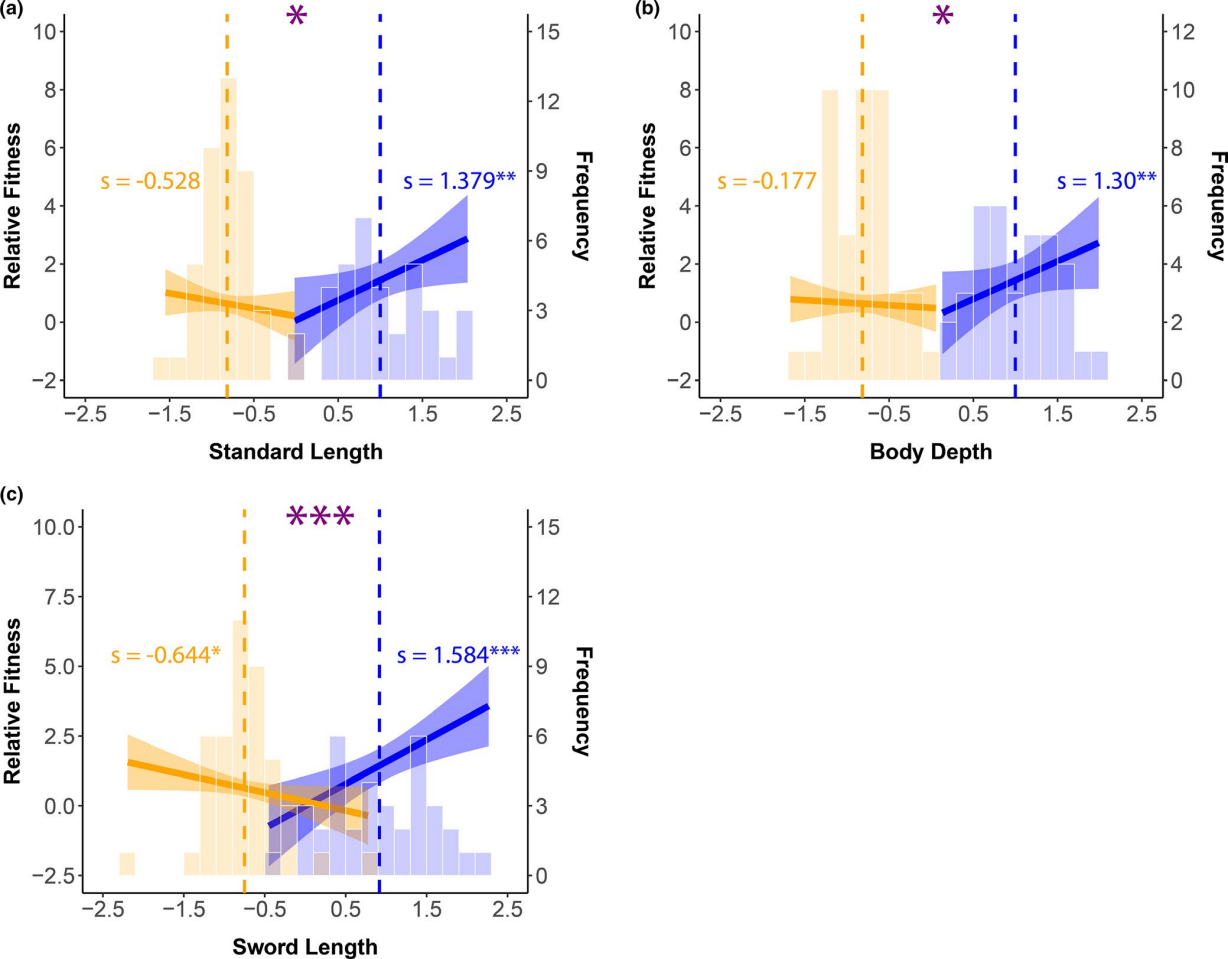
Selection differentials for each unidimensional trait and trait distribution by ART. Solid line is the differential (*s*), and shaded regions are the 95% confidence intervals. Vertical dashed lines show the mean trait value for each ART. Histograms show the distribution of the trait values. Large purple asterisks indicate a significant difference between the slopes. Blue and orange asterisks indicate a significant slope. Orange = sneaker males, blue = courter males. *p ≤ .05, **p ≤ .01, and ***p ≤ .001

The differential selection coefficients for the traits male size (standard length), body depth, and sword length were all significant (Table 1A). In addition, there were significant interactions with ART for all three traits (Table 1A). When males from each ART were analyzed separately, selection differentials for all three traits for courter males were significant (Table 1A), suggesting selection for larger size, deeper bodies, and longer swords (Figure 3). However, the selection differentials for sneaker males were only significant for sword length (Table 1A), suggesting selection on sneakers for shorter swords (Figure 3). Together, the results suggest that at least one of the ARTs is not at its optimum and that the ARTs have different optima for all three of the unidimensional traits. In addition, these results suggest that selection due to mating alone (what our data measured) is stronger in courter males than in sneaker males.

**TABLE 1:** Selection analyses on the unidimensional traits. (A) Differential selection model with both ARTs, in addition to analyses of ARTs separately for traits that had a significant interaction with ART; (B) linear and (C) quadratic selection models including all traits and interactions with ART

A	Differential (*s*)						
	SS	*F*_3,76_	*p*	Courter (s)	*p* *	Sneaker (s)	*p* *
Standard length (size)	18.049	7.834	**.008**	1.379	**.021**	−0.528	.125
ART:standard length	10.485	4.551	**.022**				
Body depth	13.550	5.703	**.015**	1.300	**.035**	−0.177	.321
ART:body depth	7.286	3.067	**.047**				
Sword length	37.155	18.413	**<.001**	1.584	**.001**	−0.644	**.028**
ART:sword length	28.071	13.911	**.002**				

B	Linear (*β*)						
	SS	*F*_7,72_	*p*	Courter (*β*)	*p* *	Sneaker (*β*)	*p* *
Standard length (size)	8.767	4.382	**.043**	2.099	**–**	−0.090	–
ART:standard length	4.061	2.030	.106				
Body depth	5.132	2.565	.076	−1.877	–	0.236	–
ART:body depth	4.269	2.134	.098				
Sword length	28.206	14.100	**.003**	1.636	**.006**	−0.684	.063
ART:sword length	19.533	9.765	**.006**				

C	Quadratic (*γ*_ii_)				
	SS	*F*_13,66_	*p*	Courter (*γ*_ii_)	Sneaker (*γ*_ii_)
Standard length (size)	0.389	0.222	.322	1.509	0.468
ART:standard length	0.061	0.035	.441		
body depth	0.399	0.227	.335	1.923	0.711
ART:body depth	0.097	0.055	.424		
Sword length	10.063	5.736	**.047**	2.392	1.716
ART:sword length	0.359	0.205	.338		

Note

*p*‐Values calculated using a resampling procedure (Lewis et al., 2011). Significant *p*‐values highlighted in bold.

Abbreviations: ART, alternative reproductive tactic; *F*, ANOVA *F*‐statistic; *p*, *p*‐Value; SS, sums of squares.

* *p* values were calculated from separate analyses by ART in cases where interactions were significant.

In the analyses of the linear (*β*) and quadratic (*γ*_ii_) selection gradients, the *β* coefficient was significant for standard length, but not for the *γ*_ii_ selection coefficients or interactions with ART (Table 1B,C). This suggests that selection on standard length detected from the differentials (see above) is direct. Inspection of the marginal effects plots suggests that the directional selection for this trait may be driven primarily by courters (see Figure S2a), similar to what was detected for the differential coefficients (see above). In the analysis of body depth, the linear (*β*) and quadratic (*γ*_ii_) selection gradients were not significant (Table 1B,C). This could suggest that selection detected by the selection differential on this trait is indirect, potentially due to a correlation with standard length. Interestingly, both the linear selection gradient (*β*) and its interaction with ART were significant for sword length (Table 1B). These results suggest that there is direct selection on sword length and that it is not in the same direction across the ARTs. Further analysis of the ARTs separately for this trait detected significant positive selection on courter males for longer swords, and a marginally nonsignificant negative selection coefficient (*s*) for sneaker males (Table 1B, see Figure S2b). Finally, as indicated by the significant quadratic selection gradient (*γ*_ii_ Table 1C) and the marginal effects plots, there may also be disruptive selection on the sword for both ARTs (Figure S2c).

**FIGURE 4:**
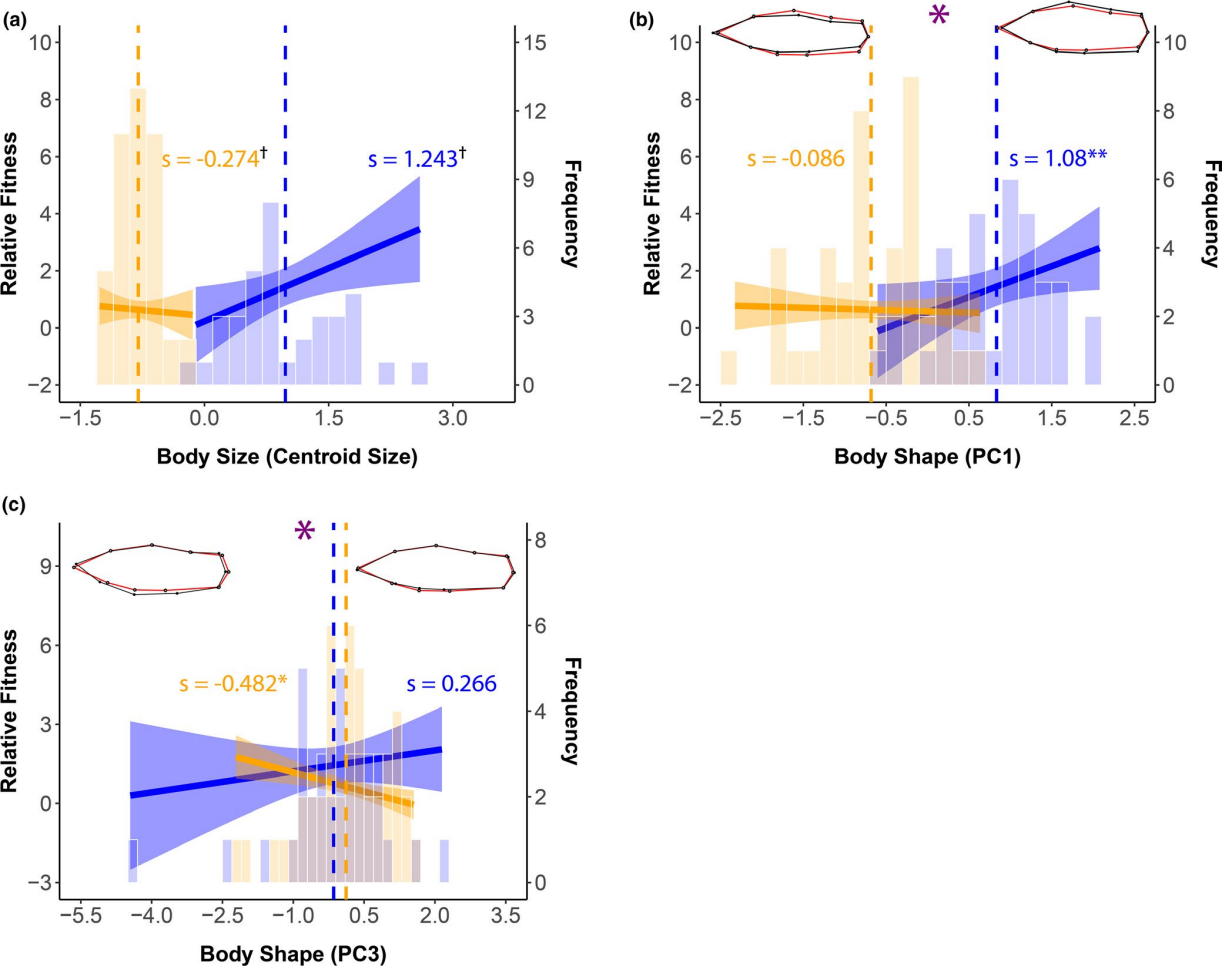
Selection differentials for each geomorphometric trait and trait distribution by ART. Solid line is the differential (*s*), and shaded regions are the 95% confidence intervals. Vertical dashed lines show the mean trait value for each ART. Histograms show the distribution of the trait values. Large purple asterisks indicate a significant difference between the slopes. Blue and orange asterisks indicate a significant slope. Wire‐frame models as explained in Figure 2. Orange = sneaker males, blue = courter males. ^†^Average slope between the ARTs is significant, we did not perform subsequent separate models in these cases because the interaction between the trait and ART was not significant (see Table 2A). *p ≤ .05, and **p ≤ .01

In our second set of analyses that considered the geometric morphometric traits, the results were similar to those that considered only the unidimensional traits. The selection differentials were significant for centroid size, the first PC axis describing body shape (PC1), and sword length (Table 2A). These results suggest that at least one of the ARTs is not at its optimum for all three of these traits. Interactions between a trait and ART were significant for body shape (PC1 and PC3) and sword length, while marginally nonsignificant for centroid size (Table 2A, Figure 4). When the ARTs were analyzed separately for body shape (PC1), we detected significant selection for deeper bodies in courter males, but not for more narrow bodies in sneaker males. For the sword, selection was for longer swords in courter males and shorter swords in sneaker males (Table 2A, Figure 4).

**TABLE 2:** Selection analyses for the geomorphometric traits, including sword length. (A) Differential selection model with both ARTs, in addition to analyses of ARTs separately for traits that had a significant interaction with ART; (B) linear and (C) quadratic selection models including all traits and interactions with ART; separate analyses by ART if interaction for trait was significant

A	Differential (*s*)						
	SS	*F*_3,76_	*p*	Courter (s)	*p* *	Sneaker (s)	*p* *
Centroid size	21.255	9.346	**.006**	1.243	–	−0.274	–
ART:centroid size	5.190	2.282	.073				
Body shape (PC1)	17.393	7.478	**.006**	1.083	**.019**	−0.086	.329
ART:PC1	11.303	4.860	**.020**				
Body shape (PC2)	3.547	1.414	.105	0.390	–	0.038	–
ART:PC2	1.966	0.783	.165				
Body shape (PC3)	3.157	1.308	.107	0.266	.170	−0.482	**.009**
ART:PC3	10.627	4.402	**.021**				
Body shape (PC4)	0.001	0.000	.486	−0.004	–	0.198	–
ART:PC4	0.743	0.294	.289				
Sword length	37.155	18.413	**<.001**	1.584	**.001**	−0.644	**.028**
ART:sword	28.071	13.911	**.001**				

B	Directional (*β*)						
	SS	*F*_13,66_	*p*	Courter (*β*)	*p* *	Sneaker (*β*)	*p* *
Centroid size	11.008	6.211	**.030**	1.141	**–**	0.415	**–**
ART:centroid size	0.737	0.416	.288				
Body shape (PC1)	5.231	2.951	.073	−0.928	**–**	0.030	**–**
ART:PC1	3.766	2.125	.107				
Body shape (PC2)	14.718	8.304	**.018**	0.860	**.036**	0.086	.288
ART:PC2	7.542	4.256	**.047**				
Body shape (PC3)	9.552	5.390	**.018**	0.501	**.048**	−0.448	**.013**
ART:PC3	15.528	8.761	**.007**				
Body shape (PC4)	1.790	1.010	.191	−0.256	**–**	0.184	**–**
ART:PC4	3.363	1.898	.128				
Sword length	29.547	16.671	**.002**	1.954	**.007**	−0.739	**.042**
ART:sword	23.837	13.449	**.003**				

C	Quadratic (*γ*_ii_)				
	SS	*F*_25,54_	*p*	Courter (*γ*_ii)_	Sneaker (*γ*_ii)_
Centroid size	14.308	8.931	**.023**	3.319	2.374
ART:centroid size	0.046	0.029	.472		
Body shape (PC1)	0.143	0.089	.370	0.326	−0.645
ART:PC1	0.742	0.463	.292		
Body shape (PC2)	3.065	1.913	.108	−0.487	−0.167
ART:PC2	0.611	0.381	.305		
Body shape (PC3)	1.492	0.931	.192	0.182	0.973
ART:PC3	2.900	1.810	.142		
Body shape (PC4)	0.083	0.052	.394	0.136	−0.063
ART:PC4	0.134	0.084	.398		
Sword length	1.050	0.655	.242	1.050	1.030
ART:sword	0.0002	0.0001	.490		

Note

*p*‐Values calculated using a resampling procedure (Lewis et al., 2011). Significant *p*‐values highlighted in bold.

Abbreviations: ART, alternative reproductive tactic; *df*, degrees of freedom; *F*, ANOVA *F*‐statistic; *p*, *p*‐Value; SS, sums of squares.

* *p* values were calculated from separate analyses by ART in cases where interactions were significant.

Further consideration of the linear (*β*) and quadratic (*γ*_ii_) selection gradients detected significant linear and quadratic selection on centroid size but no significant interactions with ART (Table 2B). These results suggest that the significant selection differential for centroid size is due to direct selection, that this selection is directional, but that differences between the ARTs were not detected (Figure S3a). Examination of the marginal effects plot for the quadratic selection gradient within each ART also suggests disruptive selection on centroid size for both ARTS, but again, differences between the ARTs were not detected (see Figure S3b). As for body shape described by PC1, the linear selection gradient was not significant, which suggests that the selection detected on this trait with the differential may be indirect. As with the unidimensional measurement of body shape (depth), PC1 may be correlated with body size. The significant linear selection gradients (*β*) and interactions with ART for body shape as described by axes PC2 and PC3 are interesting, given that only PC2 is tactically dimorphic (see above). Inspection of the marginal effects plots suggests that selection is in the opposite direction for the ARTs on these components of body shape (Figure S3c,d). Further study of the potential function of the variation in these components of body shape, and their ability to evolve differences in relation to the ARTs, would be interesting to examine. Finally, we detected significant linear selection gradient (*β*) on sword length and an interaction with ART (Table 2B), similar to the results for this trait when it was analyzed with the other unidimensional traits. Therefore, selection on the sword appears to be direct, regardless of the measures used for body size and body shape in the analyses. In addition, analyses of the ARTs separately detected selection for longer swords in courter males and shorter swords in sneaker males (Table 2B, Figure S3e).

## 4 DISCUSSION

Alternative reproductive tactics (ARTs), which involve the use of alternative mating behaviors and a suite of other traits, evolve due to intense intrasexual competition that imposes strong sexual selection (Oliveria et al., 2008). If selection can optimize reproductive success by alternative suites of traits, then we expect tactical dimorphism to evolve (Gross, 1985; Moczek & Emlen, 2000; Radwan & Klimas, 2001; Sato et al., 2004). However, little is known about whether the evolutionary constraints due to intralocus tactical conflict (IATC) can prevent traits reaching their optima within an ART. Our results suggest that body size and sword length, both tactically dimorphic traits in *Xiphophorus multilineatus*, have different optima, and one or both of the ARTs is not at its optimum for both traits, providing strong evidence of the potential for Intralocus Tactical Conflict. Body shape was also experiencing selection that was different between the ARTs, but our data did not allow us to demonstrate that the selection was direct. Finally, the selection we measured appears to be stronger for courter males than sneaker males for all of the traits except for sword length. We discuss the implications of our results in relation to the selection of these traits in this system, as well as the implications for considering intralocus tactical conflict in relation to the evolution of ARTs in general below.

Body size is the morphological trait that has been studied most extensively in relation to the ARTS in *X. multilineatus*. The ARTs are dimorphic for body size (Liotta et al., 2019, current study), and yet, our results suggest that intralocus tactical conflict could be constraining the courter males from reaching their optimal size. Variation in body size has a strong genetic influence due to variation in both alleles and copy number of the *Mc4r* gene on the *Y* chromosome (Lampert et al., 2010). However, for a trait such as male body size, it is unlikely that variation in the *Mc4r* gene is explaining all of the variation, such that numerous autosomal genes could lead to IATC. The genetic correlation across the ARTs for this trait is currently being examined and will allow us to determine the extent to which IATC is constraining this trait. It is also interesting to note that because the selection we measured did not account for the invisible fraction (males that did not survive to reproduce; Grafen, 1988), the antagonistic selection on body size we measured is not due to the differences between the ARTs in relation to the benefits and costs of reaching sexual maturity earlier (Rios‐Cardenas et al., 2018; Weinstein et al., 2019). Instead, what we measured is due to influence of sexual selection alone. This is important as it suggests that intralocus tactical conflict is an additional evolutionary mechanism beyond survival costs of maturing later that can limit the evolution of this influential trait. Finally, when body size was measured as centroid size we detected disruptive selection, selection that was not different across the ARTs. Further examination of female mate preference functions and male–male interactions has the potential to reveal a more complex role for centroid size in this system.

The results for selection on body shape were not as clear as those for body size. The selection differentials on both the unidimensional measure of body shape (body depth) and the first PC axis for the geometric morphometric measure (PC1) were significant, but the selection gradients for both measures of this trait were marginally nonsignificant. One possible explanation is that the selection on this aspect of body shape is indirect, due to correlation with another trait. We have shown previously that body size and shape are phenotypically correlated across both courters and sneakers (Liotta et al., 2019). However, we also presented two theoretical juvenile development pathways for body shape (see Figure 5 in Liotta et al., 2019) that could both explain this correlation. In one, body shape differences between the ARTs are attributable to differences in ages at sexual maturity alone. In the other theoretical pathway, the differences between the ARTs in body shape are due to different developmental paths, in which case body shape could potentially evolve independently of size. To tease apart these two scenarios, future studies of the developmental trajectories of both body shape and body size across the ARTs are planned.

Another interesting result from the analyses of selection on body shape is that, similar to the results for body size, the selection differentials for body depth and PC1 were significant for courter males but not sneaker males. Given that deeper bodies increased the size of the signal to female sail‐fin mollies (MacLaren et al., 2004), selection for deeper bodied courter males in *X. multilineatus* is not too surprising, as this trait could have increased mate attraction. We had, however, expected selection for more fusiform shapes in the sneaker males to allow for less drag and improved prolonged swimming for males using the sneak‐chase mating behavior. One possible explanation for the apparent lack of selection on body shape in the sneaker males is their behavioral plasticity, which could buffer selection for a body shape that is optimal for use with either behavior (Abbott et al., 2019b). The correlation between body shape and propensity to use sneak‐chase we detected previously (Liotta et al., 2019) supports this interpretation. More recent work detected this same relationship in laboratory‐reared males with limited experience in addition to wild‐caught males, providing further evidence to suggest that the relationship is genetically influenced and not learned (Liotta et al., in prep). In addition, the fact that we could detect selection for an overall deeper body shape in the courter males, which are fixed for courtship, lends further support to this hypothesis. Finally, there is the interesting possibility of using a comparative study to further test the hypothesis that behavioral plasticity is buffering selection on body shape in this system. Two closely related species have evolutionarily lost large males size and are fixed for sneak‐chase behavior (*X. continens* and *X. pygmaeus*, Ryan & Causey, 1989, Morris et al., 2005). We predict that body shape in both of these species will be experiencing stronger selection than what we detected in the *X. multilineatus* sneaker males.

Given that body shape is a multidimensional trait and the PC axes that we examined are a composite of multiple characteristics of, in this case, body shape, they are not independent characters with specific biological meanings (Klingenberg & Monteiro, 2005; Kuchta & Svensson, 2014; Mitteroecker & Bookstein, 2011). While this can complicate the interpretation of selection on individual PC axes, the benefit is that each axis reflects linear combinations of traits and therefore includes correlational selection between the different elements of shape described by each PC axis (Kuchta & Svensson, 2014). As for direct selection on PC2, it would be interesting to further explore the potential function of a more upturned head and the elongation of the caudal peduncle. Variation in these aspects of body shape has been previously detected in relation to habitat differences (e.g., Franssen et al., 2013; Piñeros et al., 2015), but to our knowledge has not been examined in relation to mating behaviors. However, these morphological variations seem to be in accordance with the use of unsteady swimming (Langerhans, 2009; Langerhans & Reznick, 2010), predominantly used by courters, as opposed to a more steady swimming favored by the sneak‐chase behavior used by sneakers. Finally, aspects of body shape may aid courter males in maneuverability or even fast starts (Blake, 2004; Langerhans, 2009; Oufiero, Jugo, et al., 2014; Webb, 1994), allowing the execution of rapid back‐and‐forth turns used in both courtship display and aggressive interactions with other males.

The sword on *Xiphophorus* fishes has long been a trait of interest in evolutionary biology (Darwin, 1871) as it illustrates the importance of trade‐offs between sexual selection and natural selection (Basolo, 1990a, 1990b; Darwin, 1871; Hernandez‐Jimenez & Rios‐Cardenas, 2012; Oufiero, Meredith, et al., 2014). We provide the first evidence of direct selection on this trait in a wild population, in addition to the potential for IATC to be constraining its evolution. Across many *Xiphophorus* species, females are known to prefer males with longer swords (Basolo, 1990a, 1990b, 1995; Rios‐Cardenas & Morris, 2011, although see Rosenthal et al., 2002), which would explain the direct positive selection on sword length in the courter males. Selection on the sword for sneaker males was also significant, but negative. The potential for locomotor and metabolic costs of possessing a sword (Basolo & Alcaraz, 2003; Royle et al., 2006; although see Oufiero, Jugo, et al., 2014) could explain the negative selection in males that primarily use coercive mating behaviors. The differences in selection on the sword across the ARTs suggests that Intralocus Tactical Conflict could be constraining the courter males from evolving longer swords, while at the same time constraining the sneaker males from evolving shorter swords. Therefore, even though sword length is dimorphic for the ARTs, neither ART is at its optimum for this trait.

Further evidence that intralocus tactical conflict may be constraining the evolution of the sword comes from comparative studies of sword length across species of Northern Swordtails fishes. There are two independent losses of the large male morphs in this clade (*X. continens* and *X. pygmaeus*; Morris et al., 2005), which would reduce or eliminate intralocus tactical conflict. As predicted based on the selection on sword length in sneaker males we detected, loss of the larger males would reduce or eliminate Intralocus Tactical Conflict, leading to the evolution of shorter swords. In both species, all males have evolutionarily lost the sword (Morris et al., 2005; Rauchenberger et al., 1990; Ryan & Causey, 1989). An additional comparative study of swordtail fishes could determine whether the presence of more distinct alternative mating tactics and antagonistic selection, such as detected here for the sword in *X. multilineatus*, could help explain variation in sword length across species. The sword index for *X. multilineatus* males, whether considering the average across all males (0.4 ± 0.02 *SE*, *N* = 80), or for just the courter (0.5 ± 0.02), or sneaker males (0.32 ± 0.01) in the current study, is less than the average for *X. nezahualcoyotl* (0.56) and *X. montezumae* (1.0; laboratory‐reared males; Rauchenberger et al., 1990), species without discrete ARTs. We suggest that the evolution of this hallmark trait for sexual selection may be influenced by variation across species in intralocus tactical conflict, in addition to variation in the strength of female mate preference (Rosenthal et al., 2002) and predation (Basolo & Wagner, 2004; Hernandez‐Jimenez & Rios‐Cardenas, 2012).

Understanding tactically antagonistic selection based on data from a wild population adds to the growing body of research suggesting that IATC is constraining ARTs from reaching their fixed phenotypic optima. ARTs are ubiquitous across taxa, and a better understanding of IATC will ultimately elucidate evolutionary processes and patterns we see across species with ARTs and other polymorphisms. It is also clear that IATC needs to be considered when explaining variation in sexually selected traits both within and across taxa. Finally, given that loss of an ART is associated with speciation across a range of species (ant species, Oettler et al., 2010; the side‐blotched lizards, Corl et al., 2010; northern swordtail fishes, Morris et al., 2013), further study of how these losses release traits from intralocus tactical conflict could help explain the rapid evolution of the remaining ART (West‐Eberhard, 1986), which could lead to a speciation event (Abbott, et al., 2019; Bonduriansky, 2011; Bonduriansky & Chenoweth, 2009).

## CONFLICT OF INTEREST

The authors have no conflicts of interest to declare.

## AUTHOR CONTRIBUTION

**Melissa Nena Liotta:** Conceptualization (equal); Data curation (lead); Formal analysis (lead); Methodology (equal); Writing‐original draft (lead); Writing‐review & editing (equal). **Jessica Abbott:** Conceptualization (equal); Data curation (supporting); Formal analysis (supporting); Funding acquisition (equal); Methodology (equal); Writing‐review & editing (equal). **Molly Morris:** Conceptualization (equal); Data curation (supporting); Formal analysis (supporting); Funding acquisition (equal); Methodology (equal); Writing‐review & editing (equal). **Oscar Rios‐Cardenas:** Conceptualization (equal); Data curation (supporting); Formal analysis (supporting); Funding acquisition (equal); Methodology (equal); Writing‐review & editing (equal).

## Supporting information

Fig S1

Fig S2

Fig S3

Appendix S1

## Data Availability

Data are available on Dryad at https://doi.org/10.5061/dryad.ns1rn8prz.
