# Supplementary figures and images for: Antagonistic selection on body size and sword length in a wild population of the swordtail fish, Xiphophorus multilineatus: Potential for intralocus tactical conflict
Source: Ecol Evol. 2021 Mar 20;11(9):3941–55. doi: 10.1002/ece3.7288 (PMC8093718; doi:10.1002/ece3.7288)

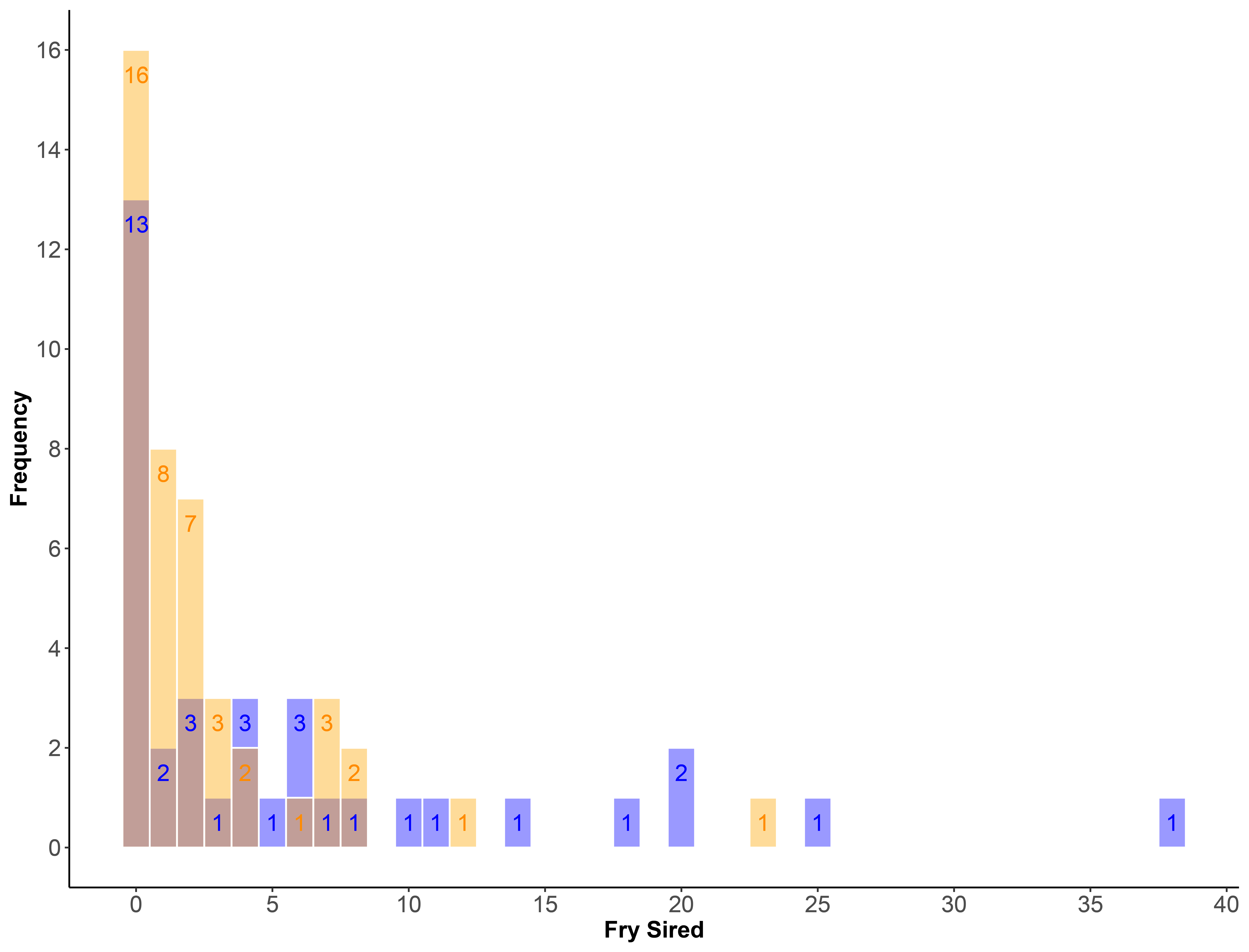

Supplement: Supplementary file 1 — Fig S1 [file ECE3-11-3941-s004.jpg]

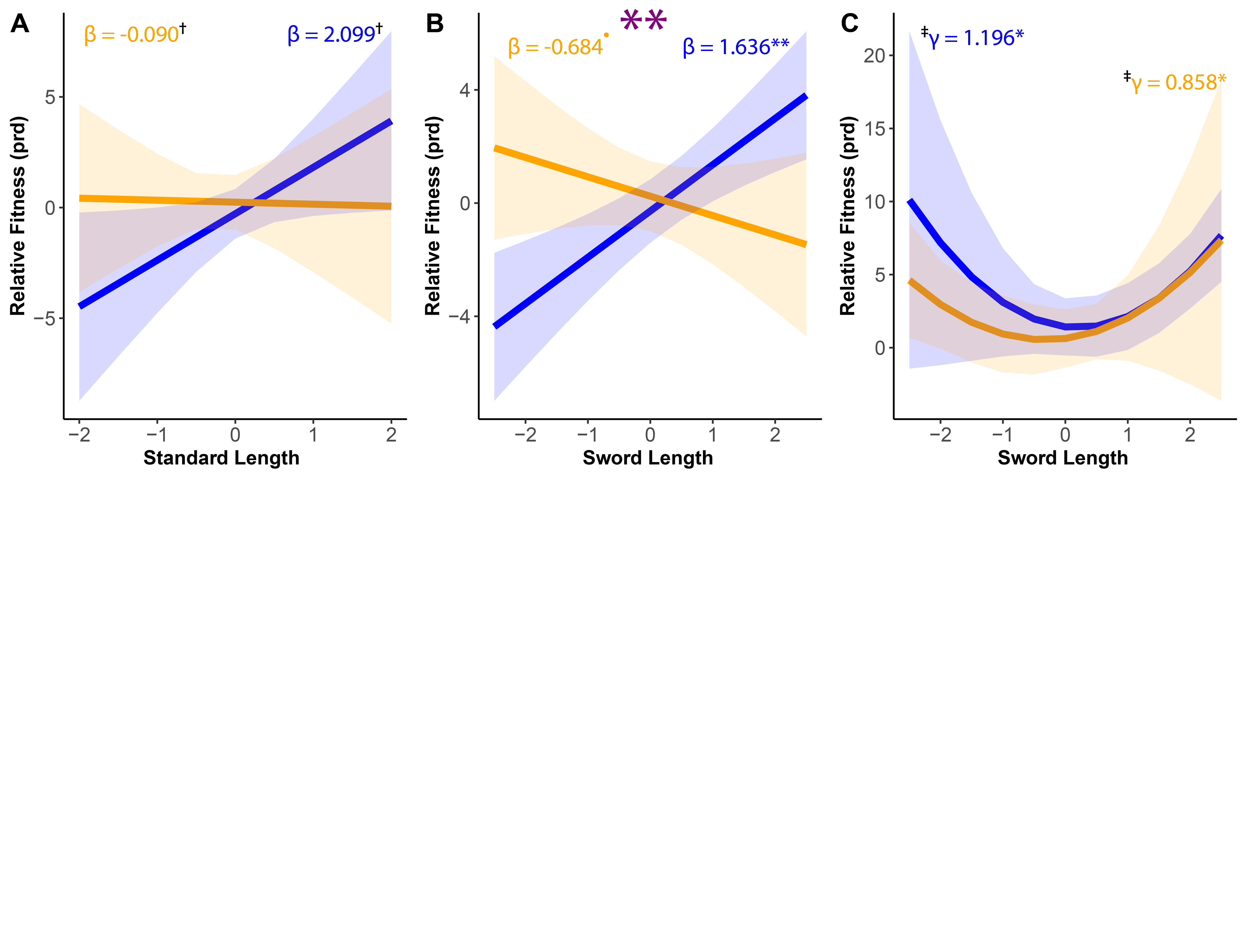

Supplement: Supplementary file 2 — Fig S2 [file ECE3-11-3941-s003.jpg]

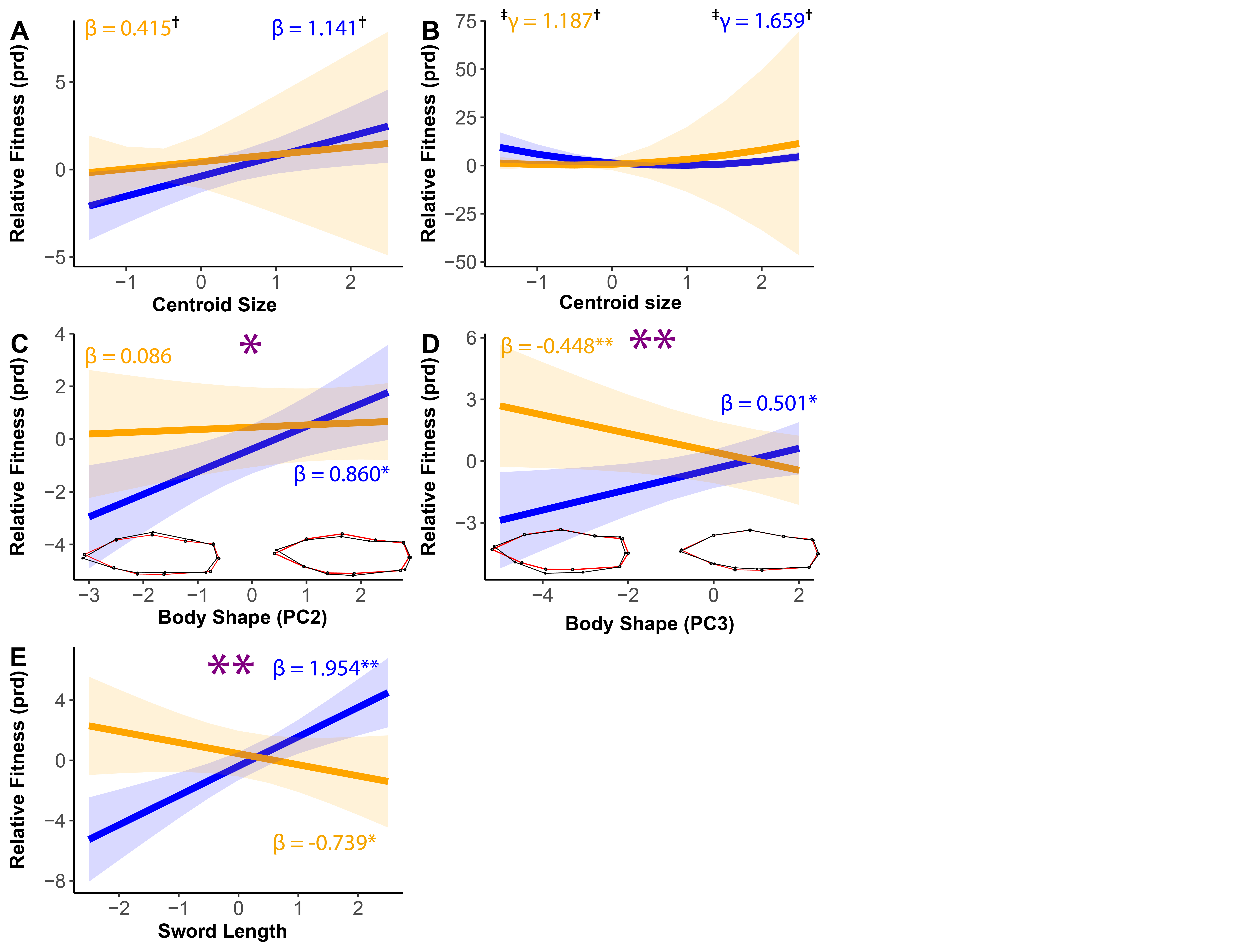

Supplement: Supplementary file 3 — Fig S3 [file ECE3-11-3941-s002.jpg]
