# Supplementary material for: Antagonistic selection on body size and sword length in a wild population of the swordtail fish, Xiphophorus multilineatus: Potential for intralocus tactical conflict
Source: Ecol Evol. 2021 Mar 20;11(9):3941–55. doi: 10.1002/ece3.7288 (PMC8093718; doi:10.1002/ece3.7288)
Supplement: Supplementary file 4 — Appendix S1 [file ECE3-11-3941-s001.docx]

**APPENDIX**

*Microsatellite Genotyping*

Primer stock was made to include a 2 µM concentration of each primer (forward and reverse) for each multiplex, except for *KonD6* in MIX1, which had a 1 µM concentration due to its intense peaks exhibited in test plates. The PCR cocktail for each sample consisted of a final volume of 10 µl and the following: 2.55 µl of RNAse-Free Water (Type-it Microsatellite PCR kit, Qiagen, Hilden, Germany), 5.0 µl of 2x Type-it Microsatellite PCR Master Mix (Qiagen, Hilden, Germany, final concentration 0.06x), 1.45 µl of the primer stock (final concentration 0.29 µM), and 1 µl of DNA.

Figure S1. Frequency of how many males in each ART (courters = blue, sneakers = orange) sired a certain number of fry. Numbers within each bin correspond to the number of sires.

Figure S2. Marginal effects plots for traits in the unidimensional analysis showing the effect of each response variable on the predicated values (prd) of relative fitness when all other traits are held constant. Solid line is the selection gradient (*β* or γ_ii_), and shaded regions are the 95% confidence intervals. Large purple asterisks indicate a significant difference between the slopes. Blue and orange asterisks indicate a significant slope. Orange = sneaker males, blue = courter males. † Average slope between the ARTs is significant, we did not perform subsequent separate models in these cases because the interaction between the trait and ART was not significant (see Table 1B-C). ‡ Quadratic coefficients visualized here are on the original scale but are reported in Table 1 multiplied by 2 as suggested by Stinchcombe et al. 2008. ^•^ P = 0.06, *P ≤ 0.05, **P ≤ 0.01, and ***P≤ 0.001

Figure S3. Marginal effects plots for traits in the geomorphometric analysis showing the effect of each response variable on the predicated values (prd) of relative fitness when all other traits are held constant. Solid line is the selection gradient (β or γ), and shaded regions are the 95% confidence intervals. Large purple asterisks indicate a significant difference between the slopes. Blue and orange asterisks indicate a significant slope. Orange = sneaker males, blue = courter males. Wire-frame models as explained in Figure 2. † Average slope between the ARTs is significant, we did not perform subsequent separate models in these cases because the interaction between the trait and ART was not significant (see Table 2B-C). ‡ Quadratic coefficients visualized here are on the original scale but are reported in Table 2 multiplied by 2 as suggested by Stinchcombe et al. 2008. ^•^ P = 0.06, *P ≤ 0.05, **P ≤ 0.01, and ***P≤ 0.001
